# Green Synthesis of Silver Nanoparticles: Structural Features and *In Vivo* and *In Vitro* Therapeutic Effects against *Helicobacter pylori* Induced Gastritis

**DOI:** 10.1155/2014/135824

**Published:** 2014-08-06

**Authors:** Muhammad Amin, Sadaf Hameed, Asghar Ali, Farooq Anwar, Shaukat Ali Shahid, Imran Shakir, Aqdas Yaqoob, Sara Hasan, Safyan Akram Khan, Sajjad-ur-Rahman

**Affiliations:** ^1^Department of Chemistry, University of Sargodha, Sargodha 40100, Pakistan; ^2^College of Pharmacy, Salman bin Abdulaziz University, Al-Kharj 11942, Saudi Arabia; ^3^Department of Physics, University of Agriculture, Faisalabad 38040, Pakistan; ^4^Deanship of Scientific Research College of Engineering, King Saud University, P.O. Box 800, Riyadh, Saudi Arabia; ^5^Center of Excellence in Nanotechnology Research Institute, King Fahd University of Petroleum and Minerals, Dhahran 31261, Saudi Arabia; ^6^Institute of Microbiology, Faculty of Veterinary Sciences, University of Agriculture, Faisalabad 38040, Pakistan

## Abstract

This study evaluates *in vivo* and *in vitro* anti-*Helicobacter pylori (H. pylori)* efficacy of silver nanoparticles (Ag-NPs) prepared via a cost-effective green chemistry route wherein *Peganum harmala* L. seeds extract was used as a reducing and capping agent. The structural features, as elucidated by surface plasmon resonance spectrophotometry, transmission electron microscopy, and powder X-ray diffraction spectroscopy, revealed the Ag-NPs synthesized to be polydispersed in nature and spherical in shape with 5–40 nm size. A typical Ag-NPs suspension (S_5_), with size being 15 nm, when tested *in vitro* against forty-two local isolates and two reference strains, showed a considerable anti*-H. pylori* activity. In case of *in vivo* trial against *H. pylori* induced gastritis, after oral administration of 16 mg/kg body weight of S_5_ for seven days, a complete clearance was recorded in male albino rates. In comparative time-killing kinetics, S_5_ exhibited dose- and time-dependent anti-*H. pylori* activity that was almost similar to tetracycline and clarithromycin, less than amoxicillin, but higher than metronidazole. Furthermore, S_5_ was found to be an equally effective anti-*H. pylori* agent at low (≤4) and high pH with no drug resistance observed even up to 10 repeated exposures while a significant drug resistance was recorded for most of the standard drugs employed. The present results revealed the potential of the synthesized Ag-NPs as safer bactericidal agents for the treatment of *H. pylori* induced gastritis.

## 1. Introduction

Nanotechnology has become a rapidly growing field of research because of the unique properties and broad range of applications of nanobioactive materials in different areas of industrial and biochemical sciences [1, 2]. Of the nanomaterials, the silver nanoparticles (Ag-NPs) have attracted a great deal of scientific interest due to their vast applications in the field of medicine [3], catalysis [4], and electronics [5–7]. So far, many methods including physical, chemical, and biochemical methods are being employed for the preparation of Ag-NPs. In this regard, chemical methods are discouraged [8] as they involve the use of noxious reducing agents and solvents [9]. With the growing awareness about the safe and clean environment the use of green approaches in synthetic processes is gaining much importance [10]. Therefore, several methods have been developed to avoid the use of synthetic/chemical reducing and stabilizing agents [2]. To facilitate the medicinal applications of Ag-NPs, it is fascinating that the materials used as aids in synthesis should be biocompatible [11]. Therefore, plant based reducing and stabilizing agents are being preferred over the chemical methods [12].

It has been established that* H. pylori* is the major cause of functional dyspepsia, chronic mucosal inflammation in the stomach and duodenum, peptic ulcer, and many other gastroduodenal abnormalities [13]. It is a small curved Gram-positive and microaerophilic bacterium which is fastidious to grow under ordinary growth medium [13]. Triple therapy has been recommended for the complete eradication of* H. pylori* [14]. Presently, the major concern around the world in medical science is that* H. pylori *has developed resistance against the standard antibiotics being used in the clinical practices [15]. Therefore, there is a need for the development of novel antimicrobial agents possessing superior effectiveness against* H. pylori *with reduced toxicity for human cells. So far, metals including zinc [16], bismuth [17], and silver-NPs [11] have been searched for their* in vitro* antibacterial activity against* H. pylori*. A zinc-based mucosal protective agent, polaprezinc [18], and various salts of bismuth have been recognized as effective antiulcer and anti-*H. pylori* drugs and indexed in various pharmacopeias [19]. Because of well-defined antimicrobial activities and potential role in wound healing [20] Ag-NPs can be explored as a possible treatment for gastrointestinal related* H. pylori* infections.

*Peganum harmala* L., commonly known as wild rue, is a medicinally important plant from the family Nitrariaceae. In the Soon Valley, Punjab, Pakistan, it is locally known as “*harmala*.” The seeds of* Peganum harmala *(*P. harmala*) contain several alkaloids [21] and other phytochemicals that attribute various medicinal properties to this multipurpose plant [21]. The seeds of this plant possess antibacterial activity against drug-resistant bacteria [22], smoke from the seeds kills algae, bacteria, intestinal parasites, and molds, and the roots are used to kill lice and insects [21]. Cytotoxic activity against the tissues of liver and kidney at very high dose of 150 g/kg of body weight in rats has been reported [22]. However, at a dose range of 75–100 g/kg body weight in rats, moderate liver and kidney toxicity was observed [22].* P. harmala* extract and powdered seeds have been used in folk medicine of different parts of the world to treat colic in man and animals due to their antispasmodic effect by blocking different types of intestinal calcium channels [23].

Our earlier study showed [11] that Ag-NPs had broad and selective* in vitro* antimicrobial activity against the antibiotic-resistant and antibiotic-susceptible strains of* H. pylori*. In the present study, we prepared highly stable Ag-NPs via a cost-effective green chemistry route using* P. harmala *seeds extract as a reducing and capping agent. The main objective of this study was to investigate the* in vitro* and* in vivo* anti-*H. pylori* activity of the synthesized Ag-NPs in a systemic infection model to fill the gap between* in vitro* characterization and clinical trials. This is perhaps the first time that we have evaluated the* in vivo* efficacy of Ag-NPs against* H. pylori* induced ulcer using an animal model.

## 2. Materials and Methods

### 2.1. Preparation of Green Reducing Agent

*P. harmala* seeds were collected from Kanhati Garden, Soon Valley, Punjab, Pakistan, during the months of September-October 2010 and were identified and authenticated by Dr. Amin Shah, Department of Biological Sciences, University of Sargodha, Sargodha, Pakistan. A specimen of the seeds was also kept at the Herbarium of the University of Sargodha, Sargodha, Pakistan. Dried seeds (100 g) were ground by a conventional coffee grinder and extracted with methanol for 48 h using a Soxhlet extractor. Afterwards, the residue was reextracted with the same fresh solvent; both of the extracts were pooled and concentrated to dryness with a rotary evaporator (45°C). The crude concentrated* P. harmala suspension* (PH-sus) was preserved at −4°C and used for further experiments.

### 2.2. Synthesis of Ag-NPs under Optimized Conditions and Structural Feature

To an appropriate amount of PH-sus, prepared above, AgNO_3_ (90 mL, 1.0 mM) was added dropwise under vigorous stirring by the use of a magnetic stirrer-cum-hotplate at ambient to 100°C. The inputs of the reagents used in the synthesis are given in Table 1.

The experiment was repeated by using 2.0 and 3.0 mM AgNO_3_ separately in order to study the effect of concentration of the silver salt on Ag-NPs characteristics. The effect of reaction time (10–180 min), medium-pH (4–10), and temperature (ambient to 100°C) on the synthesis was studied by surface plasmon resonance (SPR) spectral measurements. The Ag-NPs suspension was washed several times with Nanopure water in order to free the NPs from any unreacted silver salt.

### 2.3. Surface Plasmon Resonance (SPR) Spectroscopic Measurements

Ag-NPs suspension was appropriately diluted with water in order to obtain the SPR spectrum within 200–700 nm range by using Pharma Spec UV-1700 (Shimadzu, Tokyo, Japan) UV-Vis spectrophotometer by taking extract blank as a reference. Average size of the NPs was calculated from SPR measurements according to the already reported method [24] using the equation

(1) $$\begin{array}{c} d=\frac{1}{L_{2}}\cdot L_{n}\left(\lambda \text{SPR}-\frac{\lambda^{{}^{\circ}}}{L_{1}}\right), \end{array}$$

where *λ*° = 512, *L*_1_ = 6.53, and *L*_2_ = 0.0216.

### 2.4. X-Ray Diffraction Analysis

Powder X-ray diffraction (P-XRD) spectra of silver-NPs were recorded on Bruker D8 Discover (Germany) diffractometer using monochromatic Cu K*α* radiation (*λ* = 1.5406 Å) operating at 40 kV and 30 mA. The data for the lyophilized NPs were collected over a 10–80° 2*θ* range. The size of nano-crystallite was calculated from the full width at half maxim (FWHM) of the most intense peak of the spectrum by the use of Debye-Scherrer equation (*D* = 0.9*λ*/*β*cos⁡*θ*).

### 2.5. Transmission Electron Microscopy (TEM)

An ultrasonically dispersed sample of the solution of NPs (one drop) was placed on a carbon grid, dried at room temperature in clean environment, and TEM images were obtained by using JEM-1200EX (JEOL, Japan) microscope at an accelerating voltage of 120 kV. The average size of NPs was calculated measuring the diameter of about 140 particles by using Origin 7.5 software.

### 2.6. FT-IR Spectroscopy

Infrared spectra of the Ag-NPs were recorded by reflectance method on Perkin-Elmer Spectrum 100 FT-IR.

### 2.7. *In Vitro* Anti-*Helicobacter pylori* Activity

Forty-two local isolates [11, 16] and two standard strains of* H. pylori, *NCTC 11637 and NCTC 11638, obtained from the National Health Protection Agency, London, were used in this study.* In vitro *anti*-H. pylori* activities of the NPs sample S_5_, amoxicillin (AMX), tetracycline (TET), clarithromycin (CLT), and metronidazole (MNZ) were determined by agar dilution method according to already reported method [11, 16]. All the isolates were transferred and inoculation procedures were conducted under the BSL-III safety cabinet throughout.

### 2.8. *In Vivo* Anti-*Helicobacter pylori* Activity

*In vivo* anti-*H. pylori* activity of the S_5_ was studied in male albino Wistar rats of 72–112 days having average weight of 295 ± 4.1 g. For this purpose, the rats were infected by oral administration of* H. pylori* reference strain and then treated with different doses of silver nanoparticles. An already reported method with slight modification was adapted in this study [11].

The rats were maintained in the separate animal house facilities and acclimatized for one week prior to the experiment. All the experiments were conducted in accordance with the ethical protocols and guidelines provided by the Ethical Committee for Experimental Animals, University of Sargodha, Pakistan. The rats were kept on fasting for 18 h and offered oral dose with 0.5 mL of brain heart infusion broth (BHIB) containing a fresh diluted culture of* H. pylori* strain (3.6 × 10^8^ CFU/animal; NCTC 11637). The control rats were inoculated with blank medium. Inoculation was repeated after three days and the *H. pylori* was monitored in the gastric lavage through microscopic examination after one week postinoculation. After confirmation the rats were segregated into various groups. Different concentrations of the S_5_ were prepared as per dosage scheme of the already reported method [11] and administered orally as suspension in edible oil. The dosage scheme was selected by keeping in view the* in vitro *MICs of S_5_ experiment. The dosing was started 7 weeks after* H. pylori *inoculation and continued twice a day for 7 days. On the 3rd day after final dose, the rats were killed and counted for viable* H. pylori* counts. This was done by grinding the tissue of the removed stomach between the frosted ends of the glass slides and inoculating on the Columbia agar plates under microaerophilic conditions. The colonies of* H. pylori *were counted and the clearance rate was determined by Dunnett's method and Fisher's exact test.* P* values below 0.05 were considered statistically significant.

### 2.9. Time-Killing Kinetics of Silver Nanoparticles

This study was done according to the standard guide for assessment of antimicrobial activity using time-killing kinetics procedure [25]. The guidelines provided by CLSI for the determination of time-killing kinetics were adapted throughout this investigation. Cultures of* H. pylori* (NCTC 11637) freshly grown over Colombia agar plates were serially adjusted to initial cell concentration of 1 × 10^6^ CFU/mL. BHIB broths with 2.5% foetal bovine serum (10 mL) containing S_5_ at concentrations of 1.0 *μ*gmL^−1^, 2.0 *μ*gmL^−1^, 4.0 *μ*gmL^−1^, 8.0 *μ*gmL^−1^, and 16 *μ*gmL^−1^ were inoculated with 10 *μ*L of the fresh bacterial culture and shaken at 37°C in a microaerophilic atmosphere. Aliquots (100 *μ*L) were removed during shaking at various time points (0, 1, 2, 3, 6, 9, and 24 h). The aliquots were 10-fold serially diluted in Brucella broth and a 50 *μ*L portion of each of them was seeded over the plates of BHIA supplemented with 7% defibrinated horse blood. The plates were kept under microaerophilic atmosphere at 37°C for 72 h. Colonies were counted after 72 hours of incubation in a microaerophilic atmosphere and rates of killing were determined in duplicate by measuring the reduction in viable bacteria (log⁡_10_⁡ CFU/mL).

### 2.10. Effects of pH on Bactericidal Activity of Silver Nanoparticles

Silver-NPs (S_5_) in the concentration range of 2.0–16 *μ*g mL^−1^ were added to BHIB with 7% defibrinated horse blood. The pH was adjusted to 7, 6, 5, and 3 by using 1N urea solution as appropriate and the bacterial suspension (NCTC 11637; 10 *μ*L) was seeded into it. Urea was added to control the lethality of acidic pH to* H. pylori*. Cultures were incubated and aliquots were collected at various time points (0, 2, 3, 4, 6, 7, 9, and 24 h). The aliquots were serially diluted to 10-fold with saline and 10 *μ*L of the diluted sample was placed on Colombia agar plate with 7% defibrinated horse blood. The plate was incubated at 37°C under microaerophilic conditions for 72 h and colonies of* H. pylori *were counted.

### 2.11. Determination of Resistance Development in* H. pylori* Exposed to Silver Nanoparticles

*H. pylori* strain NCTC 11637, adjusted to a culture density of approximately 10^6^ CFU/mL in Brucella broth supplemented with 2.5% FBS, was exposed to serial twofold dilutions of S_5_ (2.0–16 *μ*g/mL), AMX (0.0125–1.0 *μ*g/mL), and TET (8.0–64 *μ*g/mL). After the incubation of the bacteria at 37°C under microaerophilic conditions for 24 h, the culture was examined for any visible growth of bacteria. The culture that attained turbidity comparable to that of the untreated culture in the presence of the highest level of the test agent was further exposed to increasing concentrations of the test agent. These procedures were repeated for three times. The fluctuations of the MIC in the course of the repeated exposure of the bacteria to the test agent were determined.

## 3. Results and Discussion

### 3.1. SPR Measurements

The SPR absorption was found to be extremely dependent upon the size, shape, interparticle distance, and the surrounding media of the Ag-NPs [26]. Thus, the shape and the size of the NPs can easily be optimized by the use of SPR spectra.

**Figure 1:**
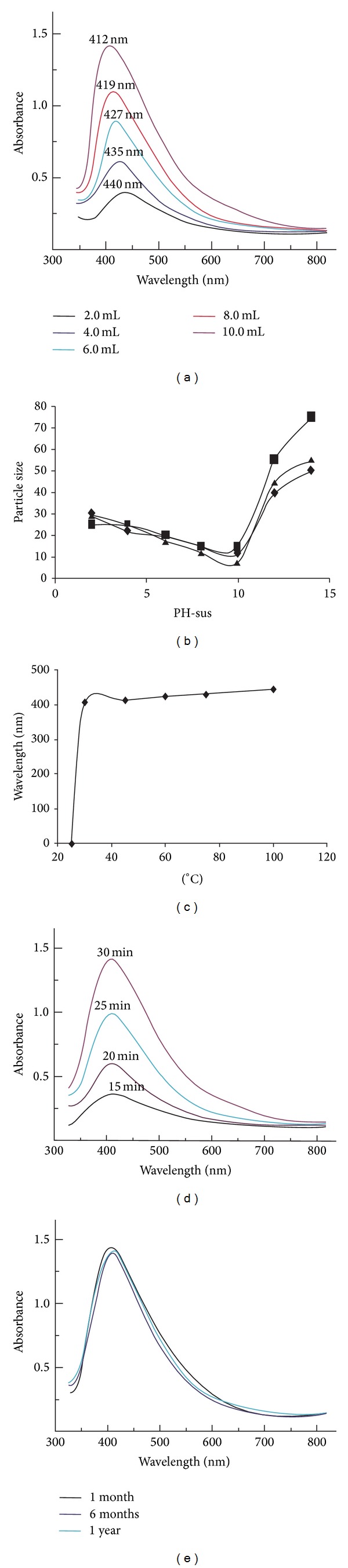
(a) Effect of PH-sus on the SPR of typical sample S_5_, (b) variation of the size of the silver-NPs with PH-sus using different techniques, SPR (*♦*), P-XRD (■), and TEM analysis (▲), (c) variation of SPR with temperature (S_5_), (d) effect of time on the synthesis of sample S_5_, and (e) stability of Ag-NPs versus time.

For the synthesis of a typical sample S_5_, PH-sus (10 mL) suspension was added to silver nitrate solution (90 mL; 1 mM aqueous) and heated at 45°C. After 30 min a colour change (yellowish brown) was observed in the reaction mixture which showed the formation of silver-NPs [11, 12]. SPR spectra were found to be highly sensitive to the concentrations of PH-sus (Figure 1(a)). For an illustration, an increasing amount of the PH-sus (2.0–10 mL) caused a significant shift in the UV-Vis region of the spectrum (440–412 nm). This signifies the role of reducing agents (PH-sus) in tailoring the size and shape of NPs as the spectrum can experience a shift dependent upon the particle size, shape, and the surrounding medium [26]. Therefore, it was observed that beyond an optimum concentration of the PH-sus the size of the particles is so increased; that is, it ceased to be in nanolimits [27]. Such observations can be used in calculating the size of the particles from SPR measurements [2]. The size of silver-NPs calculated by various techniques in this study is presented in Figure 1(b).

The effect of temperature on the synthesis of typical sample S_5_ is shown in Figure 1(c). No band appeared in SPR spectrum of the reaction carried out below 25°C, while a broad peak of very low intensity (not shown in Figure 1(c)) was observed at 406 nm for the colloidal suspension obtained after heating at 30°C for 30 min. However, for the yellowish brown colloidal suspension obtained after 30 min of stirring at 45°C, an SPR band at 412 nm suggested the formation of Ag-NPs [26]. Further increase in temperature resulted in broader peaks until the peak intensity became constant beyond 100°C. The optimum temperature for the synthesis of typical sample was found to be 45°C. Beyond this temperature the solution becomes more viscous perhaps due to the aggregation and increased size of the nanoparticles [11].

The reaction-stirring-time is an important factor affecting the optimization of SPR peak intensity. A very clear and intense absorption band (Figure 1(d)) appearing at 412 nm after 30 min of stirring, for a brown colloidal solution, indicated the presence of spherical NPs [1]. Further stirring up to 180 min, at the same conditions, resulted in a viscous dark brown solution having SPR at 499 nm. It was, therefore, asserted that an optimum time of 30 min was required for the completion of reaction.

Another important factor is pH that affects the synthesis,* in vivo* stability, and anti-*H. pylori *activity of Ag-NPs [28]. Therefore, it is equally important that the silver-NPs must be stable even at low pH (≤4) values as they are desired to be administered in the stomach. In order to obtain SPR at 412 nm the optimum pH was found to be 4. This is in contrast to the previous studies on the synthesis of Ag-NPs [29] whereby it was found that the Ag-NPs were stable mostly at high pH and that at acidic pH clusters were obtained. This is perhaps due mainly to the presence of such phytochemicals in the PH-sus which make it an efficient reducing and capping agent even at low pH values.

The stability of the colloidal suspension S_5_ was determined periodically by taking the SPR spectra. The suspension was found to be stable for a period of more than one year and the peak intensity observed was exactly at 412 nm (Figure 1(e)).

**Figure 2:**
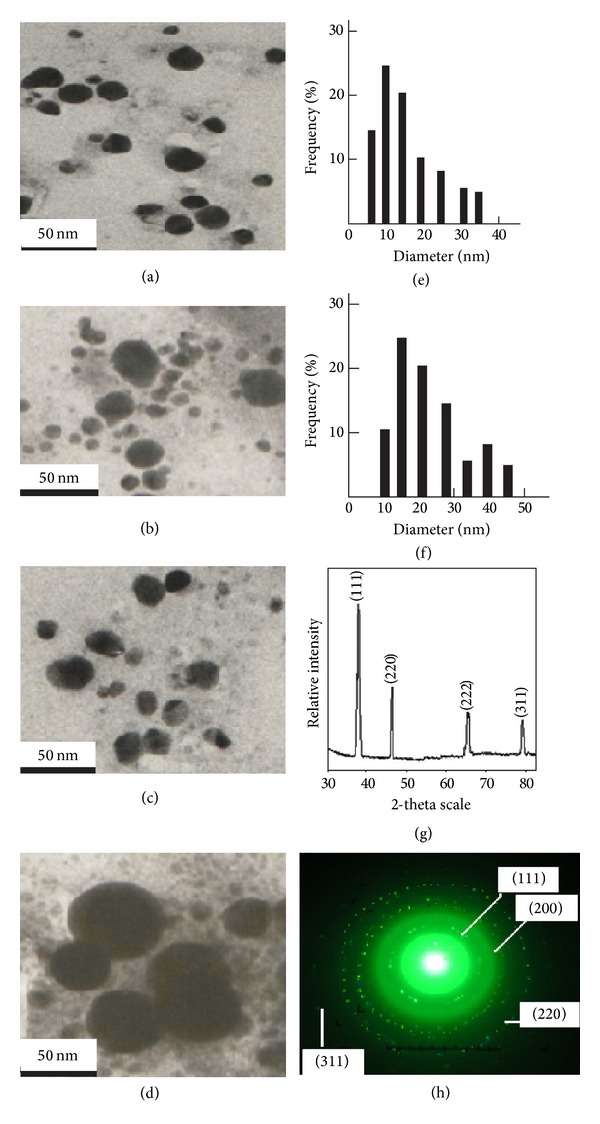
(a, e) TEM image and size distribution of S_5_, (b, f) TEM image and size distribution of S_4_, (c) TEM image of S_3_, (d) TEM image of S_2_, and (g) P-XRD pattern of S_5_ and SAED pattern of S_5_.

In order to investigate the effect of silver nitrate concentration on the synthesis of Ag-NPs, the experiments were also performed by the use of 2.0 mM and 3.0 mM silver nitrate solution while keeping the PH-suspension constant and six samples (S_6_–S_11_) were synthesized. The SPR spectra of these colloidal suspensions were observed to be between 470 nm and 495 nm indicating large particle size and aggregates. A typical image of an aggregate (S_6_) is shown in Figure 2(c).

Under the aforementioned optimized conditions the synthesis reactions were assumed to be complete because no peaks due to the residues of the reducing agents were detected in the UV-Vis spectroscopy. SPR measurements have been proved to be beneficial in determining the size of the NPs [26]. The sharpness and symmetry of SPR peaks are reported to be indicative of the particle size [2]. The dependence of the SPR of NPs on particle size and wavelength was analyzed and the particle size was calculated from SPR measurements and other techniques as listed in Table 2.

### 3.2. TEM Analysis

The particle sizes, calculated by TEM measurements, of five samples (S_1_–S_5_) of silver-NPs, are presented in Table 1 whereas typical TEM images of the colloidal suspensions, S_5_ and S_4_, and their size distribution histograms are shown in Figures 2(a), 2(b), 2(e), and 2(f), respectively. The elongation of the particles, that is, the ratio of the long to the short axes, was found to be between 1.05 and 1.25, thereby suggesting the spherical nature of the particles [30]. Figure 2(a) consists of almost uniformly sized spherical NPs. The sizes of S_5_ and S_4_ were found to be 15 nm and 18 nm, respectively. These results are in accordance with the shape of the SPR bands as reported by some earlier researchers [28]. The size ranges of S_3_ and S_2_, calculated from TEM measurements, were found to be 10–25 nm and 15–30 nm, respectively (Figures 2(c) and 2(d)).

### 3.3. Powder XRD Analysis

P-XRD patterns and selected area electron diffraction (SAED) of the typical sample (S_5_) are shown in Figures 2(f) and 2(h). P-XRD consisted of intense peaks appearing at 38.2°, 44.1°, 64.3°, and 78° in the 2*θ* range of the spectrum. These spectra were indexed as (111), (200), (220), and (311) planes of face centred cubic silver with the help of the data obtained from the database of Joint Committee on Powder Diffraction Standards file number 04-0783. In the spectrum (111) facet reflections were found to be most intense as compared to the rest of the peaks. This feature attributes special bactericidal properties to the silver-NPs [9]. The full width at half-maxim (FWHM) of the (111) face was calculated and the average size of the samples (S_1_–S_5_) calculated by the use of Debye-Scherer equation is shown in Table 2. On the basis of the smallest size, spherical shape, and the aforementioned features, the tests of the studies were conducted only for the typical sample S_5_. The patterns appearing in SAED were characterized as (111), (200), (300), and (200) face-centered cubic (fcc) plane of the crystal structure.

### 3.4. FT-IR Spectroscopy of Silver Nanoparticles

**Figure 3:**
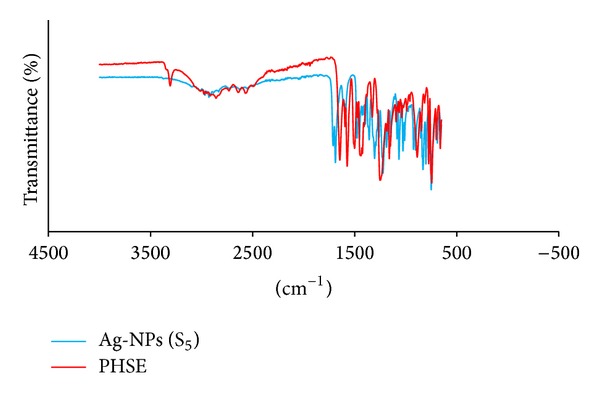
(a) FT-IR spectra of PH-sus and (b) FT-IR spectra of Ag-NPs (S_5_).

FT-IR measurements were carried out in order to identify the potential of biomolecules in PH-sus responsible for reduction and capping of silver nanoparticles. The characteristic peaks appearing in the spectrum of PH-sus (Figure 3(a)) at about 3600, 1763, and 1334 cm^−1^ are characteristic of *ν*(OH), the C–O, and C=O stretching modes of the carboxylic acid group. The bands appearing at 1669 and 1535 cm^−1^ were assigned to amide I and amide II bands, respectively, which may arise due to carboxyl stretch and N–H deformation vibrations in the amide linkages of some proteins present in them [5]. The disappearance of *ν*(OH) (Figure 3(b)) in the spectra of silver nanoparticles is consistent with the rearrangement and deprotonation of the O–H and some other groups in the PH-sus to be involved in the stabilizing of silver nanoparticles [5, 6].

### 3.5. *In Vitro* Anti-*Helicobacter pylori* Activity

The growth inhibition activities of S_5_ against* H. pylori* reference strains (NCTC-11637 and NCTC-11638) and antibiotic-resistant and antibiotic-susceptible isolates of* H. pylori* are listed in Table 3. It was also found that the anti-*H. pylori *activity of S_5_ against antibiotic-resistant isolates was nearly comparable to those against antibiotic-susceptible isolates.

### 3.6. *In Vivo* Anti-*H. pylori* Activity

**Figure 4:**
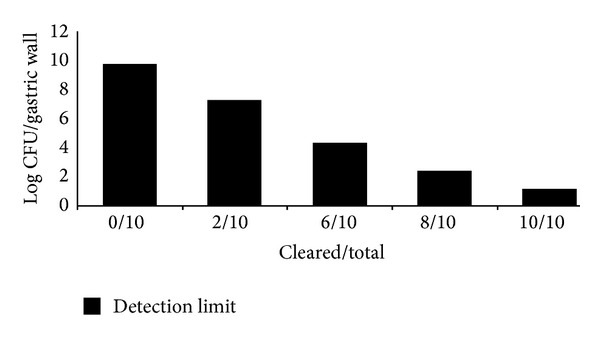
*In vivo* therapeutic efficacy of silver nanoparticles.

All of the vehicle-treated and control rats were maintained with gastric* H. pylori *at a level of approximately 1 × 10^6^ CFU. The application of S_5_ was found to be efficacious in curing ulcer by inhibiting* H. pylori. *Complete clearance was obtained at a dose of 16 mg/kg of body weight (Figure 4).

### 3.7. Time-Killing Kinetics of Silver Nanoparticles

**Figure 5:**
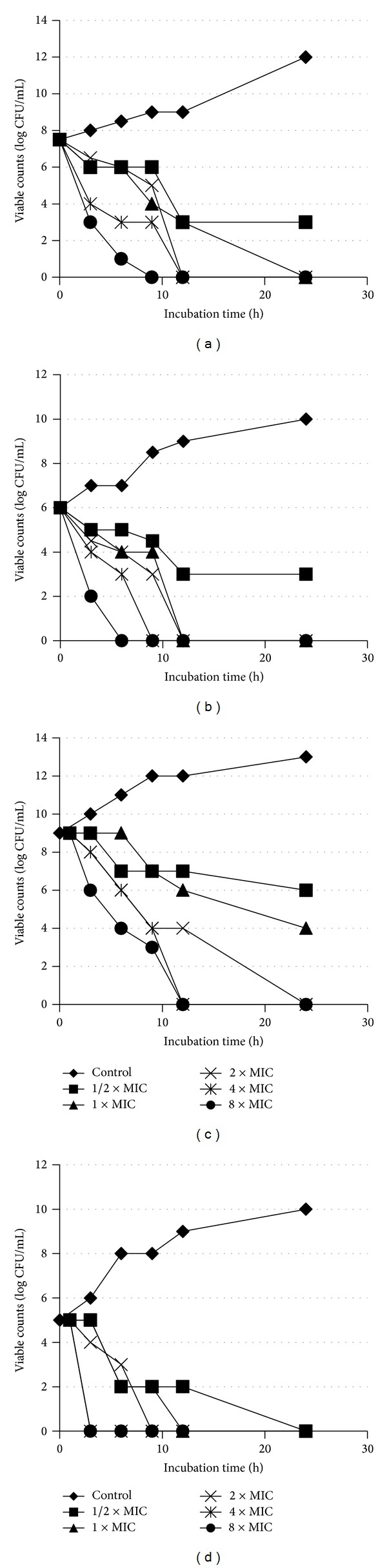
Time- and dose-dependent killing curves for (a) silver nanoparticles, (b) tetracycline, (c) metronidazole, and (d) amoxicillin, against* H. pylori* strain NCTC 11637.

Time- and dose-killing curves of S_5_, TET, AMX, and MNZ against* H. pylori *strain NCTC 11637 as function of viable bacterial counts versus incubation time are shown in Figures 5(a)–5(d). The curves for the rest of the strains/isolates are not shown. S_5_ exhibited (Figure 5(a)) bactericidal effects at concentrations of 4.0 *μ*gmL^−1^, 8.0 *μ*gmL^−1^, and 16 *μ*gmL^−1^. It was found that, at 16 *μ*gmL^−1^ dose, S_5_ was found to be effective for eradicating* H. pylori* strains within time range of 9 h followed by 8.0 *μ*gmL^−1^ dose in 12 h time. However, after 24 h contacts time NPs at most of the concentration range (4.0–16 *μ*gmL^−1^) showed potent bactericidal effect against the tested* H. pylori* strains. At a lower concentration of 2.0 *μ*gmL^−1^, no influence or a slight decrease in CFU/mL was noted. S_5_ generally exhibited rapid killing effect demonstrating concentration- and time-dependent bactericidal activity. At 4.0 *μ*gmL^−1^ the inhibition occurred at 12 h after the addition of* H. pylori* suspension to the concentrations. These results at higher concentrations of Ag-NPs were found to be almost comparable to the clarithromycin-resistant isolates of* H. pylori* (data not shown).

Amongst the standard antibiotics used in this study TET (Figure 5(b)) exhibited anti-*H. pylori* activities almost comparable to those of S_5_ (effective concentrations 4.0–16 *μ*gmL^−1^; 9.0–24 h) whereas AMX (Figure 5(c)) showed the best bactericidal activities (effective dose 0.25–4.0 *μ*gmL^−1^). MNZ (Figure 5(d)) was found to be less potent than S_5_ and almost no* H. pylori* strain was found to be susceptible to it. However, at higher concentration (1024 *μ*gmL^−1^) a slight decrease in CFU/mL was noted.

### 3.8. Effect of pH on the Anti-*H. pylori* Activity of Silver Nanoparticles

**Figure 6:**
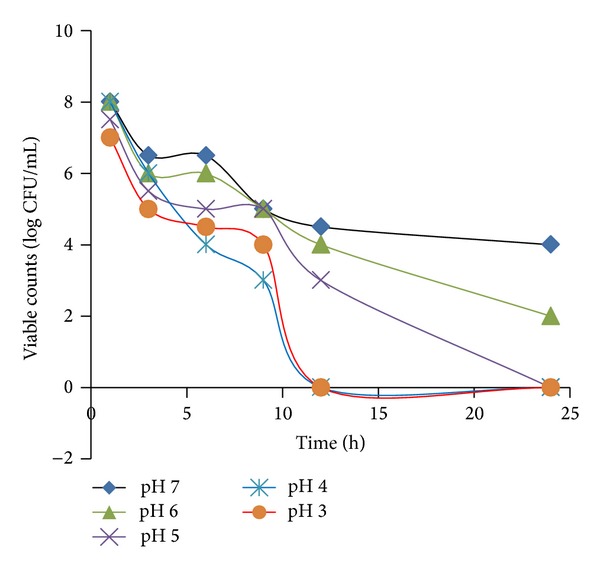
Effect of medium pH on the anti-*H. pylori* activities of silver nanoparticles against NCTC 11637.

The effect of medium-pH on the anti-*H. pylori* activities of S_5_ against* H. pylori *strain NCTC 11637 at MIC 16 *μ*gmL^−1^ is presented in Figure 6. It was found that the bactericidal activity of the S_5_ was not affected by the medium-pH (3–7). S_5_ (concentration 16 *μ*gmL^−1^) at pH 3 and pH 5 exhibited potent bactericidal effects and the viable bacterial counts reduced rapidly at 7 h after the NPs contact. However, the complete eradication was found to be possible after about 12 h time. The complete eradication (the time at which viable counts become zero) at pH 5 was possible after 24 h.

### 3.9. Development of Resistance in* H. pylori* to the Silver Nanoparticles

**Figure 7:**
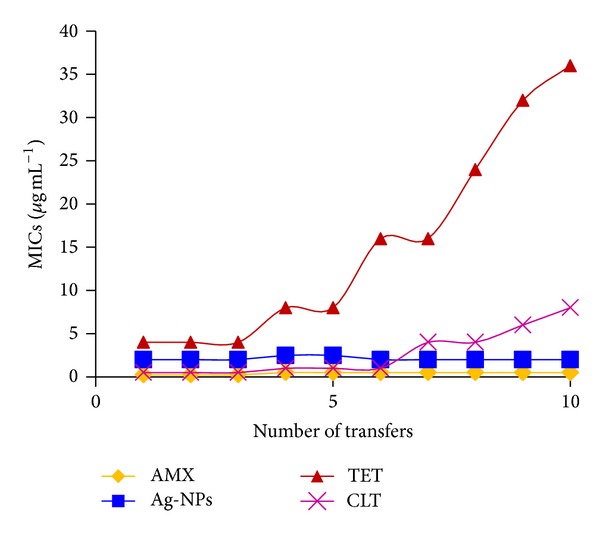
Resistance developments in* H. pylori* strain NCTC 11637 after repeated exposure to silver nanoparticles and standard antibiotics.

The fluctuations of the MICs in the course of the repeated exposure of the bacteria to the silver-NPs (S_5_) and other test drugs are shown in Figure 7. No significant changes to the MICs of S_5_ and AMX were found. However, a growing drug resistance was observed in the case of TET and MNZ after the fifth repeated exposure.

The use of solvents during drug synthesis leads to residual solvents in the final products and causes negative impact on health and the environment [31]. Currently, it is highly recommended that the drug substances should be synthesized by solvent and noxious chemical free methods [2]. The present investigation revealed that the* P. harmala *seeds extract not only reduced the silver ions but also efficiently caped the synthesized NPs at least up to more than two years. The role of capping agents in the synthesis of NPs formulations is of immense importance and, recently, in an* in vivo* study, it was demonstrated that the capped silver-NPs possessed enhanced antimicrobial activities than the uncapped ones [31].

Synthesis of Ag-NPs, using green chemistry principles whereby some plant extracts can be used as reducing and capping agents, has received special attention due to maintaining an aseptic environment during the environment-friendly process [12]. Green synthesis of NPs has novelty and innovation with regard to variation in particle size, shape, and synthesis conditions.

In our previous* in vitro* study [11] we have found that all of the tested clinical isolates (*H. pylori*) were susceptible to silver-NPs synthesized by the use of a green method. The current study was aimed at facilitating the* in vivo *clinical manifestations of silver-NPs. In the current investigation it was found that an oral administration of 16 mg/kg body weight of S_5_ resulted in the complete clearance of gastric infection induced by 3.6 × 10^8^ CFU/animal of* H. pylori* inoculums.* In vitro* time-killing kinetics showed that viable counts were reduced to zero 12 h after bacterial contact with 8.0 *μ*gmL^−1^ of S_5_. However, the same bacterial eradication was achieved in 8 h by administering 16 *μ*gmL^−1^ of S_5_. This shows that* in vitro* susceptibilities are in consistency with the* in vivo* findings. Some discrepancies have previously been reported between the* in vitro* antibacterial activities and the clinical efficacies of several antibacterial agents towards the eradication of* H. pylori* related infections [32]. Some earlier researchers [33] have reported that an ingestion up to 16 mg of silver is well tolerated in humans. Furthermore, it has low toxicity and minimal side effects when ingested since at most 2–4% is retained in tissues after absorption by the body [34]. Development of antimicrobial agents for the eradication of multidrug-resistant (MDR) microbes is a challenge for the synthetic chemists [11]. It has been reported that the efficacy of the triple therapy regime is decreasing to unacceptable levels (i.e., ≤80%) [11, 12] due to the antibiotic resistance in* H. pylori* [11–15]. Almost similar resistance patterns were found in the present research in case of TET and CLA. However, no drug resistance was found in* H. pylori* after prolonged exposure to S_5_. Metallic silver and its compounds have been used as antimicrobial agents and disinfectants because of their mild toxicity to humans [3]. However, with the development of synthetic antimicrobials for the treatment of infectious diseases, the use of silver in the clinical setting had been restricted solely to the topical use [3]. Due to the emergence of drug-resistant bacteria, there has been a resurgence of the promotion of silver-NPs as alternate antibiotics [8]. Therefore, silver-NPs find extensive applications in the field of medicine as anti-inflammatory agents [35], in wound healing [3, 11], and as antimicrobial agents against various classes of Gram-positive and Gram-negative bacteria [20].

*In vivo *anti-*H. pylori* activity of silver-NPs may be conferred from the small size, preferential penetrability to the target site, and potent wound healing properties [11]. One possible explanation for the accordance between* in vitro* MICs and* in vivo* efficacy of S_5_ may be its stability under acidic conditions. Some metals complexes have been reported for possessing bactericidal activities against* H. pylori *[11, 16]. Among these bismuth compounds like bismuth subsalicylate, bismuth subcitrate, and ranitidine bismuth citrate have officially been recommended as a part of triple therapy [36]. Due to toxic effects of bismuth on human cells [17, 35] some other metals and their NPs including Ag-NPs can be explored as a possible treatment for treatment of gastrointestinal and* H. pylori* related infections.

## 4. Conclusions

A green method has been reported for the synthesis of Ag-NPs using* P. harmala* L. seeds extract as reducing and capping agent. It was found that the size and the shape of the Ag-NPs could be tailored by optimizing the reaction temperature, time, and pH of the media. The optimized extract pH value, temperature, and molar ratio of the reactants improved the size and the shape of Ag-NPs. The adopted method is compatible with green chemistry approaches as the* P. harmala* L. seeds extract serves as a matrix for both reduction and stabilization of the synthesized NPs. These NPs due to biocompatibility and bactericidal potency against* H. pylori* may be exploited as an anti-*H. pylori* agent capable of replacing the existing triple and quadruple therapy regimens.

## Figures and Tables

**Table 1:** Inputs of the reagents for the synthesis of silver nanoparticles.

Sample code	PH-sus (%)	AgNO_3_ (1.0 mM; mL)	Water (%)
S_1_	20	90.0	80
S_2_	40	90.0	60
S_3_	60	90.0	40
S_4_	80	90.0	20
S_5_	10	90.0	90
S_6_	10	90∗	90
S_7_	10	80∗	90
S_8_	10	70∗	90
S_9_	10	90^†^	90
S_10_	10	80^†^	90
S_11_	10	70^†^	90

∗represents 2.0 mM AgNO_3_ solution; ^†^represents 3.0 mM AgNO_3_ solution.

**Table 2:** Size of silver nanoparticles calculated by using different techniques.

Sample code	SPR measurements		P-XRD measurements		TEM measurements (nm)
	Peak position (nm)	Calculated size (nm)	FWHM of *fcc* (111)	Calculated size (nm)	
S_1_	440	30	30	25	15–40
S_2_	435	22	25	25	15–30
S_3_	427	20	25	20	10–25
S_4_	419	15	22	15	07–14
S_5_	412	12	20	15	05–10

**Table 3:** *In vitro* minimum inhibitory concentration of S_5_ against reference strains and antibiotic-resistant and antibiotic-susceptible local isolates of *H. pylori*.

Organism	Strain/isolate	MICs (*µ*gmL^−1^) of S_5_
*Helicobacter pylori *	NCTC 11637	4.0
	NCTC 11638	8.0

	*Clinical isolates* [11, 16]	
	AMX-resistant (*n* = 08)	4.0–8.0
	AMX-susceptible (*n* = 32)	2.0–8.0
	CLT-resistant (*n* = 05)	1.0–16
	CLT-susceptible (*n* = 35)	4.0–16
	TET-resistant (*n* = 09)	2.0–8.0
	TET-susceptible (*n* = 31)	2.0–16
	MNZ-resistant (*n* = 036)	2.0–32
	MNZ-susceptible (*n* = 04)	4.0–8.0

## References

[1] Bhattacharya R, Mukherjee P (2008). Biological properties of “naked” metal nanoparticles. *Advanced Drug Delivery Reviews*.

[2] Amin M, Iram F, Iqbal MS, Saeed MZ, Raza M, Alam S (2013). Arabinoxylan-mediated synthesis of gold and silver nanoparticles having exceptional high stability. *Carbohydrate Polymers*.

[3] Leaper DJ (2006). Silver dressings: their role in wound management. *International Wound Journal*.

[4] Baruah B, Gabriel GJ, Akbashev MJ, Booher ME (2013). Facile synthesis of silver nanoparticles stabilized by cationic polynorbornenes and their catalytic activity in 4-nitrophenol reduction. *Langmuir*.

[5] P. Daizy (2010). Green synthesis of gold and silver nanoparticles using *Hibiscus rosa sinensis*. *Physica E: Low-Dimensional Systems and Nanostructures*.

[6] Thakkar KN, Mhatre SS, Parikh RY (2010). Biological synthesis of metallic nanoparticles. *Nanomedicine: Nanotechnology, Biology, and Medicine*.

[7] Montazer M, Alimohammadi F, Shamei A, Rahimi MK (2012). Durable antibacterial and cross-linking cotton with colloidal silver nanoparticles and butane tetracarboxylic acid without yellowing. *Colloids and Surfaces B: Biointerfaces*.

[8] Devaux X, Laurent C, Rousset A (1993). Chemical synthesis of metal nanoparticles dispersed in alumina. *Nanostructured Materials*.

[9] Pastoriza-Santos I, Liz-Marzán LM (2002). Formation of PVP-protected metal nanoparticles in DMF. *Langmuir*.

[10] Albrecht MA, Evans CW, Raston CL (2006). Green chemistry and the health implications of nanoparticles. *Green Chemistry*.

[11] Amin M, Anwar F, Janjua MRSA, Iqbal MA, Rashid U (2012). Green synthesis of silver nanoparticles through reduction with *Solanum xanthocarpum* L. berry extract: characterization, antimicrobial and urease inhibitory activities against *Helicobacter pylori*. *International Journal of Molecular Sciences*.

[12] Kora AJ, Sashidhar RB, Arunachalam J (2010). Gum kondagogu (*Cochlospermum gossypium*): a template for the green synthesis and stabilization of silver nanoparticles with antibacterial application. *Carbohydrate Polymers*.

[13] Dunn BE, Cohen H, Blaser MJ (1997). Helicobacter pylori. *Clinical Microbiology Reviews*.

[14] Kostamo P, Veijola L, Oksanen A, Sarna S, Rautelin H (2011). Recent trends in primary antimicrobial resistance of *Helicobacter pylori* in Finland. *International Journal of Antimicrobial Agents*.

[15] Mégraud F, Lehn N, Lind T (1999). Antimicrobial susceptibility testing of Helicobacter pylori in a large multicenter trial: the MACH 2 study. *Antimicrobial Agents and Chemotherapy*.

[16] Amin M, Iqbal MS, Hughes RW (2010). Mechanochemical synthesis and in vitro anti-Helicobacter pylori and uresase inhibitory activities of novel zinc(II)famotidine complex. *Journal of Enzyme Inhibition and Medicinal Chemistry*.

[17] Gisbert JP, Calvet X (2011). Review article: Non-bismuth quadruple (concomitant) therapy for eradication of *Helicobater pylori*. *Alimentary Pharmacology and Therapeutics*.

[18] Matsukura T, Tanaka H (2000). Applicability of zinc complex of L-carnosine for medical use. *Biochemistry*.

[19] Sweetman SC (2007). *Martindale: The Complete Drug Reference*.

[20] Fung MC, Bowen DL (1996). Silver products for medical indications: risk-benefit assessment. *Journal of Toxicology: Clinical Toxicology*.

[21] Harsh ML, Nag TN (1984). Antimicrobial principles from in vitro tissue culture of *Peganum harmala*. *Journal of Natural Products*.

[22] Mohamed AHS, AL-Jammali SMJ, Naki ZJ (2013). Effect of repeated administration of Peganum harmala alcoholic extract on the liver and kidney in Albino mice: a histo-pathological study. *Journal of Scientific & Innovative Research*.

[23] Moloudizargari M, Mikaili P, Aghajanshakeri S, Asghari MH, Shayegh J (2013). Pharmacological and therapeutic effects of harmala and its main alkaloids. *Pharmacognosy Review*.

[24] Haiss W, Thanh NTK, Aveyard J, Fernig DG (2007). Determination of size and concentration of gold nanoparticles from UV-Vis spectra. *Analytical Chemistry*.

[26] De Matos RA, Da Silva Cordeiro T, Samad RE, Vieira ND, Courrol LC (2012). Green synthesis of gold nanoparticles of different sizes and shapes using agar-agar water solution and femtosecond pulse laser irradiation. *Applied Physics A*.

[27] Vidhu VK, Aromal SA, Philip D (2011). Green synthesis of silver nanoparticles using *Macrotyloma uniflorum*. *Spectrochimica Acta A: Molecular and Biomolecular Spectroscopy*.

[28] van Hyning DL, Klemperer WG, Zukoski CF (2001). Silver nanoparticle formation: predictions and verification of the aggregative growth model. *Langmuir*.

[29] Shayesteh SF, Kolahi S, Kalandarragh YA (2013). Effect of pH on the structure and optical properties of nanoparticles embadded in PVA matrix. *Indian Journal of Pure and Applied Physics*.

[30] Prathna TC, Chandrasekaran N, Raichur AM, Mukherjee A (2011). Kinetic evolution studies of silver nanoparticles in a bio-based green synthesis process. *Colloids and Surfaces A: Physicochemical and Engineering Aspects*.

[31] Gnanadhas DP, Thomas MB, Thomas R, Raichur AM, Chakravortty D (2013). Interaction of silver nanoparticles with serum proteins affects their antimicrobial activity vivo. *Antimicrobial Agents and Chemotherapy*.

[32] Kanamaru T, Nakano Y, Toyoda Y (2001). In vitro and in vivo antibacterial activities of TAK-083, an agent for treatment of Helicobacter pylori infection. *Antimicrobial Agents and Chemotherapy*.

[33] Kim T, Kim M, Park H, Shin US, Gong M, Kim H (2012). Size-dependent cellular toxicity of silver nanoparticles. *Journal of Biomedical Materials Research A*.

[34] Kim JS, Song KS, Sung JH (2013). Genotoxicity, acute oral and dermal toxicity, eye and dermal irritation and corrosion and skin sensitisation evaluation of silver nanoparticles. *Nanotoxicology*.

[35] MubarakAli D, Thajuddin N, Jeganathan K, Gunasekaran M (2011). Plant extract mediated synthesis of silver and gold nanoparticles and its antibacterial activity against clinically isolated pathogens. *Colloids and Surfaces B: Biointerfaces*.

[36] Gisbert JP, Calvet X, O’Connor A, Mégraud F, O’Morain CA (2010). Sequential therapy for helicobacter pylori eradication: a critical review. *Journal of Clinical Gastroenterology*.

